# Greeting

**Published:** 1876-01

**Authors:** 


### Editorial.

We send this, the first number of the centennial year, to our readers with our greetings and a sincere wish that they may all enjoy a happy New Year.

With these greetings we feel it not amiss to say a word in reference to the trials and responsibilities of the journalist, and a few words of our Journal.

The labor expended in conducting a medical journal, when to the ordinary duties is added the perplexities of its financial and business detail, is a task never properly understood, particularly when to these duties is added the unceasing toil incident to the life of an active practitioner.

The urgent periodical calls for "copy" by the printer, when from other equally pressing demands upon the editor's time, "copy" has not been prepared; to return to his office at night, exhausted and worn from the day's labor, to find a half-dozen galleys of proof, with a note from the printer urging the importance of its being corrected and ready for the press by early morn; or worse still, to find when the number is issued and the printer's bill presented, that not enough has been received from subscribers to settle the bill, thus forcing him to the most unpleasant task of all, that of sending "duns" to delinquent subscribers. These are some of the trials and perplexities of the journalist when he fills the position of editor, publisher and proprietor. It is not surprising, then, that under such pressure there will occasionally creep in a mis-spelled word, or a sentence not altogether grammatic. For all these delinquencies we ask the kind indulgence of our readers. Now, a word in regard to the Journal.

This number commences the twenty-first year of the existence of the Atlanta Medical and Surgical Journal, which has, with the exception of the four years of the late war, been regularly issued to subscribers. We do not propose here a review of the work accomplished, of our shortcomings, and the vicissitudes through which we have passed; nor do we propose to note the changes which have from time to time occurred in the editorial staff, as this would be of but little interest to our readers; but propose simply to say that to-day the Journal is on a more enduring basis than at any time since its first issue.

Were we not opposed to promises from principle, we would desire to say in this connection, that we commence the labors of this the centennial year with renewed energy and with a determination to improve on the past.

